# Causal relationships between epilepsy and the microstructure of the white matter: A Mendelian randomization study

**DOI:** 10.1097/MD.0000000000040090

**Published:** 2024-11-01

**Authors:** Zhijun Xie, Zhe Chen, Yuhong Jiang, Jiaqi Yao, Pengcheng Zhang, Hang Lei, Wenfu Tang

**Affiliations:** a Department of Spleen and Stomach Diseases, The Affiliated Traditional Chinese Medicine Hospital of Southwest Medical University, Luzhou, Sichuan, China; b Department of Integrative Medicine, West China Hospital, Sichuan University, Chengdu, Sichuan, China; c Department of Thoracic Surgery, The Second Xiangya Hospital, Central South University, Changsha, Hunan, China.

**Keywords:** epilepsy, imaging-derived phenotypes, Mendelian randomization, microstructural white matter

## Abstract

To examine the causal bidirectional relationships between epilepsy and microstructural changes in the white matter (WM). A genome-wide association study meta-analysis of the International League Against Epilepsy Consortium on Epilepsy and 360 WM imaging-derived phenotypes (IDPs) from the UK Biobank was used for the analysis. Genetic correlation analyses were conducted based on summary statistics of various “IDP-epilepsy” pairs for 2-sample Mendelian randomization (MR) analysis to explore the causal relationships. We used the inverse variance weighted (IVW) method as the primary MR analysis approach, and conducted sensitivity analyses for pleiotropy and heterogeneity. Forward MR analysis revealed that alterations in the 16 WM IDPs increased the risk of epilepsy (*q* value < 0.05). Changes in the 38 WM IDPs were associated with a decreased risk of epilepsy (*q* value < 0.05). In the reverse analysis, seizures from all epilepsy types changed 5 WM IDPs, whereas seizures from juvenile myoclonic epilepsy altered 11 WM IDPs (*q* value < 0.05). This study revealed causal associations between changes in the WM microstructure and epilepsy subtypes. These findings offer new directions for early prevention and treatment of epilepsy.

## 1. Introduction

Epilepsy is a chronic central nervous system disorder that affects over 70 million people worldwide.^[1]^ It is characterized by recurrent seizure events with stereotyped changes in neural mechanisms in the brain during episodes.^[2]^ Epilepsy is a significant contributor to the years lived with disability in neurological diseases, with idiopathic epilepsy accounting for 14.1% of the disability-adjusted life-years burden among neurological conditions.^[3]^ Despite being one of the leading causes of global disability and death, approximately 50% of epilepsy cases have an unclear etiology,^[2]^ necessitating further research.

In 1888, Hughlings-Jackson proposed that epilepsy is a cerebral cortex disorder. However, advances in detection technologies have indicated that alterations in the cerebral cortex are not the sole cause of epileptic seizures. In recent years, magnetic resonance imaging (MRI) has enabled the identification of subtle changes in white matter (WM) myelination and in the number of interstitial neurons.^[4]^

Diffusion-weighted MRI and its analytical extensions, particularly diffusion tensor imaging (DTI), are currently the only noninvasive techniques capable of effectively observing and tracking WM fiber tracts in the living human brain.^[5]^ DTI precisely reveals microstructural changes in multiple regions near and far from epileptogenic foci. For example, a previous study that used MRI DTI indicated diffusion abnormalities in both superficial and deep WM in patients with temporal lobe epilepsy (TLE) and hippocampal sclerosis (HS).^[5]^ Moreover, a study employing voxel-based morphometry, an automated image analysis technique, showed that patients with TLE exhibit pathological changes in cerebral gray matter and a reduction in WM ipsilateral to the epileptic focus.^[6]^ Additionally, an observational study across various epilepsy syndromes noted abnormalities in the WM of the corpus callosum, external capsule, and cingulum in most patients with epilepsy.^[7]^ Overall, increasing evidence suggests that epilepsy is associated with changes in the microstructure of WM; however, most of this evidence is observational in nature, leaving the causal relationship between them unclear.

Mendelian randomization (MR) utilizes the naturally occurring random event of genetic variation as a powerful tool to analyze the variable risk factors in population health, providing evidence of the presumed causal relationship between modifiable risk factors and diseases.^[8]^ Since parents pass genes onto offspring through random allocation, unaffected by other external factors, MR can avoid biased associations that arise from confounding or reverse causality in observational studies.^[9]^

Recent large-scale genome-wide association studies (GWAS) of neuropsychiatric disorders coupled with brain imaging-derived phenotypes (IDPs) have allowed the exploration of causal relationships between changes in brain IDPs and neuropsychiatric disorders.^[10]^ The aim of this study was to explore the bidirectional causal effects between WM IDPs and epilepsy based on genetic variation summary statistics from the International League Against Epilepsy (ILAE) Complex Epilepsy Consortium and the UK Biobank GWAS. To do so, a 2-sample MR analysis was conducted. This study revealed a causal relationship between WM microstructure changes and various epilepsy subtypes, providing new directions for the clinical prevention and treatment of epilepsy.

## 2. Methods

In this study, the genome-wide association studies (GWAS) summary statistics for epilepsy and its subtypes were derived from the 2023 meta-analysis study conducted by the ILAE Complex Epilepsy Consortium.^[11]^ Epileptic seizures and epilepsy syndrome were classified into 3 categories according to the ILAE diagnosis: generalized epilepsy (GGE), focal epilepsy (FE), and unclassified epilepsy.^[12]^ The GWAS data for epilepsy in this study were based on data from patients of European ancestry (Supplemental Digital Content 1, http://links.lww.com/MD/N802).

GWAS summary statistics for WM IDPs were derived from genetic research on brain IDPs conducted by the UK Biobank,^[13]^ using a sample of 31,356 individuals of European descent. The UK Biobank has collected genetic and multimodal brain imaging data from approximately 50,000 people using MRI scanners and has characterized the microstructure of WM based on the obtained diffusion MRI data using 2 analytical methods: tract-based spatial statistics (TBSS) and probabilistic tractography. Both TBSS and probabilistic tractography analyses reported 6 metrics in multiple cortical regions using the DTI fitting tool and the neurite orientation dispersion and density imaging fitting tool accelerated microstructure imaging via convex optimization,^[14]^ including the fractional anisotropy (FA), mean diffusivity (MD), diffusion tensor mode (MO), intracellular volume fraction (ICVF), isotropic or free water volume fraction (ISOVF), and orientation dispersion index (OD).

Our analysis included 60 WM tracts, comprising data from 48 TBSS and 12 probabilistic tractography tracts, and incorporating 360 WM IDPs (60 WM tracts × 6 measurements), with each IDP representing the mean or weighted average of the measurements within a WM region. The data sources are listed in Supplemental Digital Content 2, http://links.lww.com/MD/N802.

### 2.1. Mendelian randomization analysis

**Figure 1.**
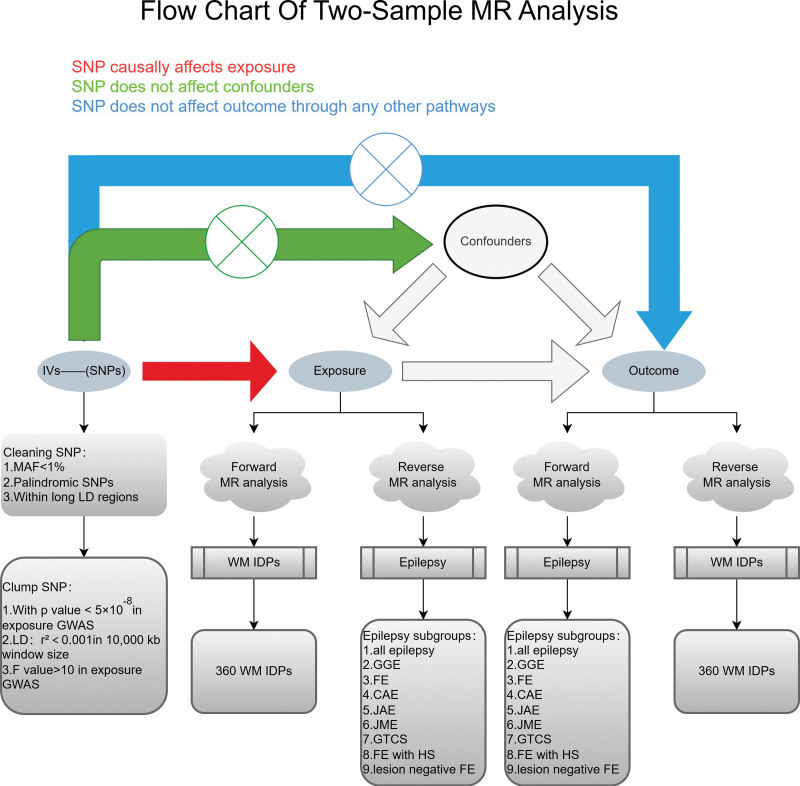
Flow chart of the MR analysis. CAE = childhood absence epilepsy, FE = focal epilepsy, FE with HS = focal epilepsy with hippocampal sclerosis, GGE = generalized epilepsy, GTCS = generalized epilepsy with tonic-clonic seizures, GWAS = genome-wide association studies, IDPs = imaging-derived phenotypes, IVs = instrumental variables, JAE = juvenile absence epilepsy, JME = juvenile myoclonic epilepsy, LD = linkage disequilibrium, MAF = minor allele frequency, MR = Mendelian randomization, SNP = single nucleotide polymorphism, WM = white matter.

The 3 fundamental assumptions of MR are as follows: genetic variants are closely associated with exposure; genetic variants are unrelated to the outcome except through exposure; and genetic variants are unrelated to any potential confounding factors.^[15]^ Therefore, we selected single nucleotide polymorphisms (SNPs) from the exposure factor GWAS data that reached a genome-wide significance level (*P* < 5 × 10^−8^). To ensure the independence of SNP instrumental variables (IVs) and avoid bias introduced by instrumental variables, we removed SNPs with linkage disequilibrium (LD) based on an *r*^2^ threshold of 0.001 within 10,000 kb as the final instrumental variables for the MR study.^[16]^ We calculated the *F* statistic (*F* = β^2^/se^2^) for each SNP corresponding to the GWAS exposure data to assess the power of the SNPs. To minimize instrument bias, only SNPs with an *F* value > 10 were considered usable IVs.^[17]^ MR analysis included previously published GWAS summary statistics. The ethics committee of each institution approved each study, and all participants provided written informed consent prior to their inclusion in the study. Each study was conducted in accordance with the principles of the Declaration of Helsinki. No additional ethical approval or informed consent was obtained from any participant. The overall study design is illustrated in Figure 1.

In this study, we conducted a bidirectional 2-sample MR analysis using the TwoSampleMR R package (https://mrcieu.github.io/TwoSampleMR).^[18]^ We divided the MR analysis into 2 categories based on different exposure and outcome factors. In the forward analysis, WM IDP was considered as the exposure factor and epilepsy as the outcome factor to investigate the influence of WM IDPs on epilepsy. In the reverse analysis, epilepsy was considered as the exposure factor and WM IDPs as the outcome factor to study the effects of epileptic seizures on various brain WM regions.

To analyze each effect, we employed 5 methods: inverse-variance weighted (IVW), MR-Egger, weighted median, weighted mode, and simple mode. Among these, the IVW method with a random-effects model was utilized as the primary MR analysis method to detect the causal effects between exposure and outcome factors. The IVW method uses an asymptotic estimation of the standard error of the causal (ratio) estimate from each variant, which provides the most robust confidence when detecting causal effects.^[19]^ The slope coefficient from the MR-Egger regression provides an estimate of the causal effect. This facilitates detection of some violations of the standard instrumental variable assumptions, and provides effect estimates that are robust to these violations.^[20]^ The weighted median method can prevent up to 50% of invalid instrumental variables.^[21]^ The weighted mode method estimates the causal effect of the subset with the largest number of SNPs by clustering the SNPs into subsets resting on the resemblance of causal effects.^[22]^ Although the simple mode is not as powerful as the IVW method, it provides robustness with respect to pleiotropy.^[23]^ Lastly, these 4 methods serve as supplementary approaches to evaluate the reliability of analyses, alongside the IVW method.

### 2.2. Sensitivity analysis

To enhance the robustness and reliability of the MR study results, we conducted sensitivity analysis. First, we assessed the heterogeneity between each SNP in the IVW method using Cochrane’s *Q* statistic, where *P* > .05 indicated no heterogeneity.^[24]^ Second, the MR-Egger regression employed the standard of residual heterogeneity and outliers to evaluate horizontal pleiotropy, where *P* > .05 indicated no horizontal pleiotropy.^[25]^ Thirdly, we use an MR-Steiger directionality test to determine the correctness of the direction of causal estimation between IDP and epilepsy.^[26]^ Leave-one-out analysis was performed to assess whether causality was driven by individual SNPs with substantial horizontal pleiotropy and to evaluate the reliability of the results.^[27]^ Finally, we employed the MR-pleiotropy residual sum and outlier (MR-PRESSO) method to identify and correct potential outliers of genetic variants with horizontal pleiotropy.^[28]^ This method serves 2 purposes: to identify potential SNP outliers and ensure that the results after removing the identified outliers are consistent with those obtained through the IVW method.

### 2.3. Reverse analysis

To investigate which brain WM regions are affected by the occurrence of epilepsy, we conducted reverse causal analysis, treating epilepsy as the exposure factor and WM IDP as the outcome factor. Odds ratios (ORs) were used to determine causal effects. The results are presented as point estimates and 95% confidence intervals.

## 3. Results

### 3.1. Causal effects of WM IDPs on epilepsy and its subgroups

**Figure 2.**
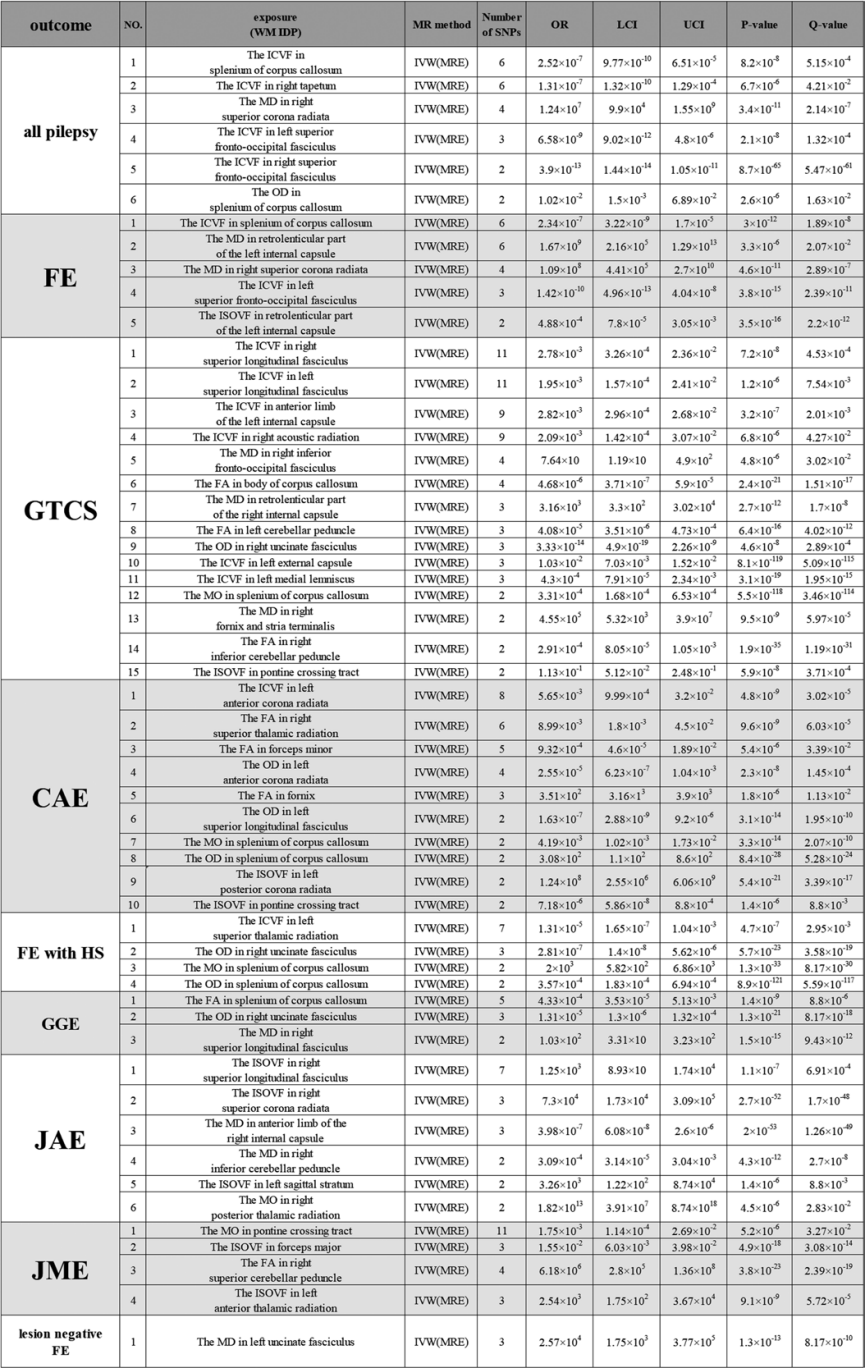
Results of MR analysis of the WM IDPs and epilepsy risk. A total of 81 WM IDPs showed a causal effect on epilepsy. The tabular file for this figure is in Supplemental Digital Content 3. CAE = childhood absence epilepsy, FA = fractional anisotropy, FE = focal epilepsy, FE with HS = focal epilepsy with hippocampal sclerosis , GGE = generalized epilepsy, GTCS = generalized epilepsy with tonic-clonic seizures, ICVF = intracellular volume fraction, IDP = imaging-derived phenotype, ISOVF = isotropic or free water volume fraction, JAE = juvenile absence epilepsy, JME = juvenile myoclonic epilepsy, LCI = lower confidence interval, MD = mean diffusivity, MO = diffusion tensor mode, MR = Mendelian randomization, OD = orientation dispersion, OR = odds ratio, UCI = upper confidence interval, WM = white matter.

**Figure 3.**
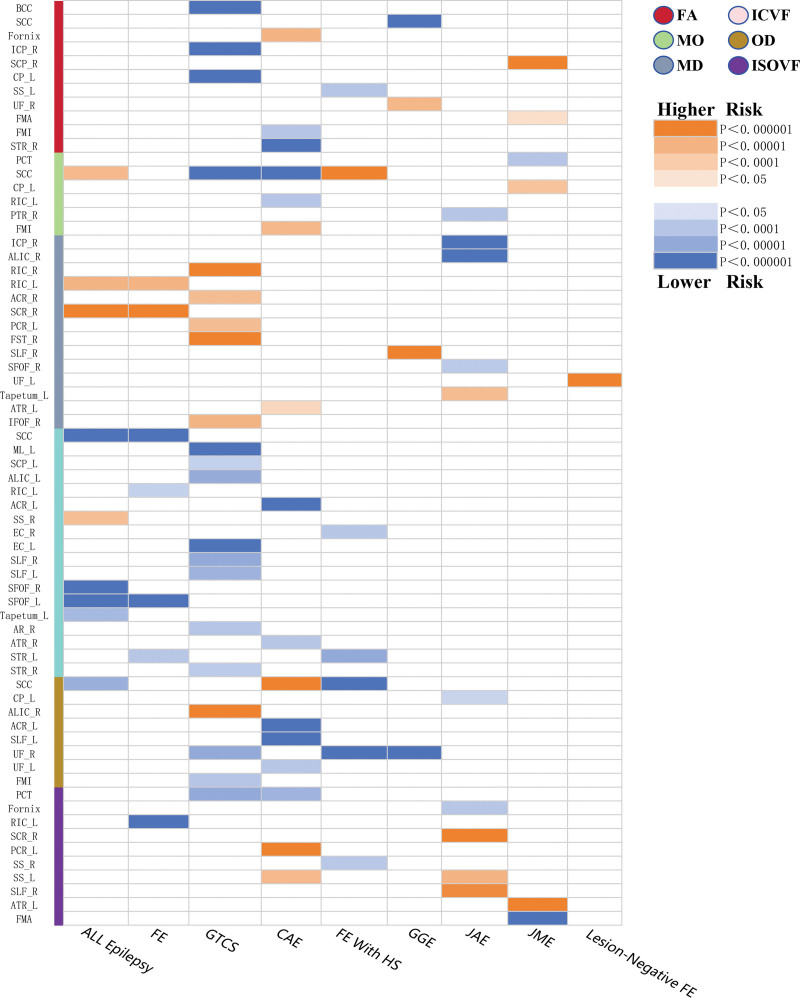
Results of WM IDPs derived from the fixed-effect IVW analysis. ACR = anterior corona radiate, ALIC = anterior limb of internal capsule, AR = acoustic radiation, ATR = anterior thalamic radiation, BCC = body of corpus callosum, CAE = childhood absence epilepsy, CP = cerebral peduncle, EC = external capsule, FA = fractional anisotropy, FE = focal epilepsy, FE with HS = focal epilepsy with hippocampal sclerosis, FMA = forceps major, FMI = forceps minor, FST = fornix and stria terminalis, GGE = generalized epilepsy, GTCS = generalized tonic-clonic seizures, ICP = inferior cerebellar peduncle, ICVF = intracellular volume fraction, IDPs = imaging-derived phenotypes, IFOF = inferior fronto-occipital fasciculus, ISOVF = isotropic or free water volume fraction, IVW = inverse-variance weighted, JAE = juvenile absence epilepsy, JME = juvenile myoclonic epilepsy, L = left, MD = mean diffusivity, ML = medial lemniscus, MO = diffusion tensor mode, OD = orientation dispersion, PCT = pontine crossing tract, PCR = posterior corona radiate, PTR = posterior thalamic radiation, R = right, RIC = retrolenticular part of internal capsule, SCC = splenium of corpus callosum, SCP = superior cerebellar peduncle, SCR = superior corona radiate, SFOF = superior fronto-occipital fasciculus, SLF = superior longitudinal fasciculus, SS = sagittal stratum, STR = superior thalamic radiation, UF = uncinate fasciculus, WM = white matter.

First, our data adhered to the following criteria: significance threshold (*P* < 5 × 10^−8^), Bonferroni correction (*P* < .05), *F* statistics (*F* > 10), and LD (*r*^2^ < 0.001, kb < 10,000). We then identified 324 IDPs that met the criteria from the initial 360 WM IDPs. Subsequent MR analyses of these 324 WM IDPs, after applying Bonferroni correction, revealed 54 WM IDPs with causal effects on epilepsy. We primarily report the results derived using the IVW method as the main analysis approach (Figs. 2 and 3; Supplemental Digital Content 3, http://links.lww.com/MD/N803), with the results from the 4 other methods presented in Supplemental Digital Content 4, http://links.lww.com/MD/N804.

WM IDPs showed the highest number of positive associations with generalized epilepsy and tonic-clonic seizures (GTCS), of which 15 were associated with the occurrence of GTCS, accounting for approximately 28% of all positive results. This was followed by 10 WM IDPs associated with childhood absence epilepsy (CAE).

In addition, most WM IDPs associated with the 9 subgroups predominantly demonstrated a relationship with reduced seizure risk, with 38 WM IDPs showing a decrease in seizure risk for their respective subgroups, accounting for 70% of the total results. Among these, WM IDPs related to reducing the risk of GTCS and CAE represented the largest proportion (22% and 13%, respectively). Notably, the left uncinate fasciculus, originating in the temporal lobe and terminating in the frontal lobe, was the sole positive finding associated with lesion-negative FE, in which an increase in MD was significantly associated with a higher risk of seizures. Interestingly, the left uncinate fasciculus showed a decrease in the orientation dispersion index, which was linked to a reduced risk of CAE seizures.

### 3.2. Sensitivity analysis

Cochran’s *Q* statistic, which incorporates the IVW test and MR-Egger regression method, indicated no significant heterogeneity among the SNPs for all positive findings (*P* > .05). The MR-PRESSO test detected no horizontal pleiotropy or apparent outliers (*P* > .05). Similar findings were obtained with the MR-Egger regression intercept analysis (*P* > .05), and the Steiger directionality test indicated no significant directionality or horizontal pleiotropy (Supplemental Digital Contents 5 and 6, http://links.lww.com/MD/N805 http://links.lww.com/MD/N806).

### 3.3. Reverse analysis

In a bidirectional MR framework (Fig. 1), we utilized epilepsy GWAS data as the exposure to conduct a reverse analysis on the outcome factor, WM IDP, and found that the occurrence of epilepsy exerts an influence on brain WM. Specifically, all epilepsy cases showed a causal effect on 5 WM IDPs (Bonferroni-corrected, *P* < .05), accounting for 31% of the total results. Juvenile myoclonic epilepsy (JME) demonstrated a causal effect on 11 WM IDPs (Bonferroni-corrected, *P* < .05), representing 69% of all results.

**Figure 4.**
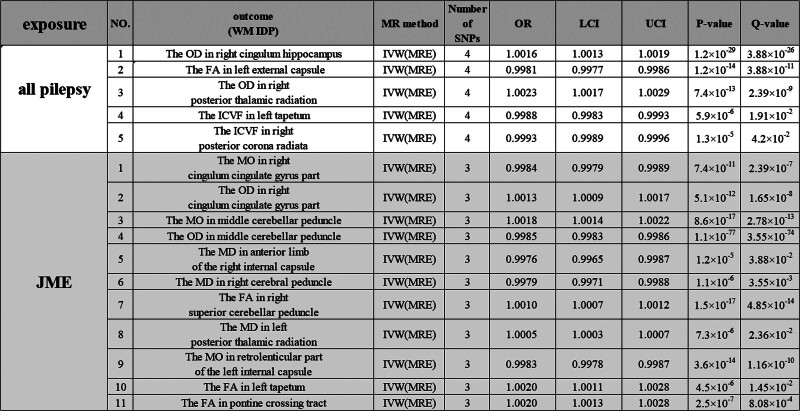
Results of MR analysis of epilepsy and WM IDP risk. Epileptic seizures can affect 21 WM IDPs. The tabular file for this figure is in Supplemental Digital Content 3. FA = fractional anisotropy, ICVF = intracellular volume fraction, IDP = imaging-derived phenotype, ISOVF = isotropic or free water volume fraction, JME = juvenile myoclonic epilepsy, LCI = lower confidence interval, MD = mean diffusivity, MO = diffusion tensor mode, MR = Mendelian randomization, OD = orientation dispersion, OR = odds ratio, UCI = upper confidence interval, WM = white matter.

We also conducted sensitivity analyses, including Cochran’s *Q* statistics, MR-Egger regression intercept analysis, and MR-PRESSO tests, none of which showed significant outliers (*P* > .05; Supplemental Digital Content 4, http://links.lww.com/MD/N804). We extracted the results obtained primarily using the IVW method (Fig. 4; Supplemental Digital Content 3, http://links.lww.com/MD/N803), and the results from the other 4 methods are displayed in Supplemental Digital Content 4, http://links.lww.com/MD/N804.

## 4. Discussion

In this study, a bidirectional 2-sample MR analysis was conducted to assess the causal relationship between microstructural changes in WM and epilepsy in patients with psychiatric disorders. In the forward MR analysis, 54 IDPs demonstrated a clear causal effect on epilepsy (Bonferroni correction, *P* < .05), primarily reducing seizure risk in the corresponding subgroups, accounting for 70% of the total results. Reverse analysis showed that epilepsy exhibited a causal effect on 5 WM IDPs (Bonferroni correction, *P* < .05), representing 31% of the total results. JME exhibited a causal effect on 15 WM IDPs (Bonferroni correction, *P* < .05), accounting for 69% of the total results.

Microstructural changes in WM had a significant causal effect on GTCS, with 15 WM IDPs showing relevance that were previously unreported in observational studies. GTCS manifests as episodic, intense, and sustained contractions of muscles on one or both sides of the body, with patients exhibiting significant impairments in mood, attention, language, and memory functions.^[29]^ Furthermore, an observational study indicated significant interstitial changes in the lymph nodes of the right thalamus and bilateral temporal lobes.^[30]^ Consequently, the majority of positive results involve neural fiber bundles, such as the cerebral peduncle, inferior cerebellar peduncle, and pontine crossing tract that transmit motor and sensory information, along with the right thalamus, corpus callosum, uncinate fasciculus, and superior longitudinal fasciculus, which are primarily involved in the regulation of memory, language, and emotional functions. Notably, an increased FA in the right inferior cerebellar peduncle and left cerebral peduncle was associated with a reduced risk of GTCS. The inferior cerebellar peduncle originates in the medullary portion of the brainstem and contains the main efferent cerebellar fibers. It primarily transmits motor and sensory information from the medulla to the cerebellum while integrating feedback from the cerebellum to the thalamus, which in turn transmits this information to cortical regions.^[31,32]^ Previous studies have found that this tract is mainly involved in the control of movement and sensation, and reflects damage occurring in motor disorders.^[33]^ The cerebral peduncle originates from the frontal, parietal, temporal, and occipital lobes and is responsible for transmitting motor and sensory information. The upper portion includes the corticospinal tract, the most significant motor pathway in the brain, which conveys motor information from the cerebral cortex to the spinal cord.^[34,35]^ FA is primarily used to observe the integrity of the WM structure and myelination, as well as the condition of nerve fiber bundles.^[36]^ An increase in FA indicates a more consistent direction of nerve fibers, suggesting a more intact and compact structure, which enhances the effective transmission of neural signals. High FA values are generally associated with a healthy state of nerve fiber bundles. Previous studies have confirmed that seizures in GTCS are associated with abnormal electrical signal transmission between the cerebral cortex and hypothalamus.^[37]^ Therefore, we hypothesized that increasing the FA of the right inferior cerebellar and left cerebral peduncles could correct abnormal electrical signal expression during epileptic seizures, thereby reducing the risk of GTCS.

Additionally, an increase in the ICVF of the superior longitudinal fasciculus (SLF), which connects the parietal, temporal, and frontal lobes of the brain, reduces the risk of GTCS. The SLF is one of the largest association fiber tracts in the brain, linking the perisylvian areas across the hemispheres and supporting cognitive function and motor control.^[38]^ Previous research has confirmed that the SLF is associated with the generation of motor hyperactivity.^[39]^ An increase in the ICVF mainly indicates an increase in the intracellular space within the nerve fiber bundles in this region, which may reflect an increase in neuronal cells, hypertrophy, or cellular edema.^[40]^ Cellular edema can affect the normal function of neurons, leading to the disruption or weakening of signal transmission. This can result in reduced or impaired mediated movements. Therefore, we believe that when the ICVF of the SLF increases, mediated hyperactivity is reduced, thereby decreasing the risk of GTCS.

Interestingly, all WM IDPs associated with an increased risk of GTCS episodes exhibited an elevated MD. An increase in MD indicates that water molecules can diffuse more freely within nerve fiber bundles, reflecting the compromised or damaged structural integrity of the nerve fiber bundles characterized by neuronal injury, axonal damage, or myelin sheath damage.^[14]^ Therefore, an elevated MD may lead to abnormalities in neuronal connectivity, affecting communication and signal transmission between neurons, thereby increasing the risk of epilepsy or exacerbating existing epilepsy symptoms. In some cases, an increase in MD may serve as an indicator of disease progression or treatment efficacy, which is important in neuroimaging, diagnosis, and monitoring.

The causal relationship between WM IDP and FE, and focal epilepsy with hippocampal sclerosis (FE with HS) is consistent with previous observational studies.^[5,6]^ Moreover, the abnormal alterations of WM IDP in these 2 subtypes are also reflected in the corpus callosum, which corroborates previous findings from observational studies investigating different epilepsy syndromes.^[7]^ This may be due to the epilepsy seizures propagating throughout the brain via myelinated tracts in the white matter, with the corpus callosum being the commissural bundle required for the generalization of epileptic seizures^[41]^; thus, alterations of WM IDP in the corpus callosum can be found in both types of epilepsy.

An increase in the diffusion tensor mode in the splenium of the corpus callosum (SCC) is associated with an increased risk of FE with HS. SCC originates in the posterior part of the brain and connects the posterior regions of the 2 cerebral hemispheres. It facilitates communication and coordination among the sensory, motor, and cognitive functions of both cerebral hemispheres, promoting the integration of information between them.^[42]^ Lesions in the SCC may lead to disconnection of the cerebral hemispheres, accompanied by cortical dysfunction, loss of consciousness, and delirium.^[43,44]^ Diffusion tensor mode (MO) provides information about the tissue microstructure, such as the orientation and connectivity of fiber bundles. It is highly sensitive to differences in WM, particularly in crossing fibers. Therefore, MO is primarily used to study the integrity of WM fiber bundles, lesions, and the progression of neurodegenerative diseases. An increase in MO indicates structural alterations and reduced WM integrity due to the loss of crossing fibers^[45,46]^ Studies have found that the majority of patients with TLE exhibit signs of HS on MR,^[47]^ widespread atrophy of the SCC, inferior fronto-occipital fasciculus, and uncinate fasciculus have been observed.^[48]^ Therefore, we believe that an increase in MO reflects the compromised integrity of the SCC and predicts an increased risk of FE with HS.

In our reverse analysis, JME influenced the structure of the WM IDP. Specifically, the pontine crossing tract (PCT) originating from the cerebral peduncle is affected, resulting in a decrease in FA. PCT transmits motor nerve fibers from one cerebral hemisphere to the contralateral spinal cord, controlling muscle activity on opposite sides of the body. FA serves as one of the output pathways from the motor areas of the cerebral cortex and plays a crucial role in the coordination and control of bodily movements.^[49,50]^ FA, in contrast, is primarily used to observe the integrity of the WM structure and myelin sheaths, as well as the extent of damage to nerve fiber bundles.^[36]^ A decrease in FA can lead to overall cognitive impairment,^[51]^ possibly due to FA reduction, indicating damage to nerve fiber bundles, anatomical abnormalities, or pathological changes. It is typically associated with WM structural damage, diseases, or abnormalities, such as brain injury, inflammatory conditions, and neurodegenerative diseases.^[37]^

Studies have indicated that GTCS in epilepsy belongs to a subset of generalized seizures that affect the brain networks across both hemispheres. They typically begin with absence epilepsy (AE) or myoclonic epilepsy that evolves into GTCS.^[37]^ Therefore, we speculate that JME, leading to damage to the PCT, may be associated with subsequent GTCS. Additionally, abnormal activity within brain networks during epileptic seizures can affect myelin sheath formation, and changes in myelin sheath thickness have been confirmed during AE seizures.^[52,53]^ Hence, we hypothesized that AE seizures may further promote abnormal changes in the myelin sheath structure and that differences in myelin sheath thickness may facilitate the progression of generalized epilepsy, as demonstrated experimentally.^[54]^ However, whether AE seizures play a driving role in the progression of generalized epilepsy requires further investigation.

In recent decades, brain connectivity research^[55]^ and intracranial neuromodulation techniques^[56]^ have played critical roles in epilepsy treatment. The thalamus, serving as an “integrative hub” within the brain, is a critical target for intracranial neuromodulation therapies. In the current study, in the anterior thalamus, superior thalamus, and posterior thalamus, IDPs, such as the ICVF in the left and right superior thalamic radiation, FA in the right superior thalamic radiation, ICVF in the right anterior thalamic radiation, MD and ISOVF in the left anterior thalamic radiation, and MO in the right posterior thalamic radiation were involved in the occurrence of focal epilepsy, generalized epilepsy with tonic-clonic seizures, childhood absence epilepsy, focal epilepsy with hippocampal sclerosis, juvenile absence epilepsy, and juvenile myoclonic epilepsy. In clinical practice, these IDPs enable more refined targeting within the thalamic neural networks of various patients with epilepsy, thereby reducing adverse reactions from neuromodulation and enhancing the precision and controllability of treatment. This, in turn, has advanced the continued development and innovation of intracranial neuromodulation techniques. Moreover, emerging evidence highlights the potential efficacy of intracranial neurostimulation interventions in pediatric populations and in patients with generalized epilepsy.^[53]^ The IDPs we identified with causal relationships with epilepsy can facilitate the selection of corresponding neural targets in clinical practice based on the specific epilepsy subtype and intracranial region involved. This enables the development of tailored intracranial neurostimulation protocols that precisely disrupt seizure propagation pathways, thereby preventing seizure recurrence.

The most substantial advantage of the current study is the Mendelian randomization design used. The use of randomly distributed genetic variations as instrumental variables (IVs) can effectively avoid reverse causality and residual confounding, which observational studies are prone to. Samples from the UK Biobank and European ancestry samples from the International League Against Epilepsy Complex Consortium were analyzed to effectively avoid biases caused by sample overlap and population stratification. IVW was used as the primary analytical method, and the robust estimation effects of each IV were determined (all *F* statistics > 10), rendering the results more reliable.

Nevertheless, this study has some limitations. Firstly, the sample was limited to individuals of European ancestry. Although this approach reduces the sample overlap, it potentially limits the generalizability of the results. Moreover, the 3 main assumptions of MR studies are stringent, and both epilepsy and microstructural alterations in WM have complex etiologies. It is possible that not all confounding factors related to exposure were accounted for, and we cannot guarantee complete elimination of bias in the causal inference estimates. Second, horizontal pleiotropy in MR studies can affect causal inference. However, the results of all the sensitivity analyses in this study suggest that the impact of horizontal pleiotropy is likely minimal. Finally, we only investigated the causal relationship between epilepsy and microstructural alterations in the WM. Further research should explore how clinical symptoms, disease severity, and duration of illness in epilepsy affect the WM microstructure.

## 5. Conclusion

In summary, we established a causal relationship between epilepsy and microstructural WM changes measured using diffusion MRI IDP metrics, based on genetic variation and MR analysis. These microstructural WM changes, which affect the risk of epilepsy onset, could potentially serve as neuroimaging biomarkers for epilepsy, aiding the early diagnosis of patients with potential epilepsy. Additionally, alterations in the microstructural WM during epileptic seizures provide potential targets for interventions in epilepsy treatment. These findings deepen our understanding of epileptic seizures and their underlying neurophysiology, offer potential targets for precise and efficient intervention in epilepsy, and provide new directions for future therapeutic strategies.

## Acknowledgments

This work was supported by a grant from the National Natural Science Foundation of China (No. 82174264).

## Author contributions

**Conceptualization:** Zhijun Xie, Zhe Chen, Yuhong Jiang, Jiaqi Yao, Pengcheng Zhang, Wenfu Tang.

**Data curation:** Zhijun Xie, Zhe Chen, Yuhong Jiang, Jiaqi Yao, Hang Lei.

**Formal analysis:** Zhijun Xie, Zhe Chen, Yuhong Jiang, Hang Lei.

**Funding acquisition:** Wenfu Tang.

**Investigation:** Zhijun Xie, Zhe Chen, Jiaqi Yao, Pengcheng Zhang.

**Methodology:** Zhijun Xie, Yuhong Jiang.

**Project administration:** Zhijun Xie, Hang Lei, Wenfu Tang.

**Resources:** Zhijun Xie, Zhe Chen, Jiaqi Yao, Pengcheng Zhang, Hang Lei.

**Software:** Zhijun Xie, Pengcheng Zhang, Hang Lei.

**Supervision:** Zhe Chen, Jiaqi Yao, Pengcheng Zhang, Wenfu Tang.

**Validation:** Zhijun Xie, Zhe Chen, Yuhong Jiang, Jiaqi Yao, Pengcheng Zhang.

**Visualization:** Zhijun Xie, Zhe Chen, Yuhong Jiang.

**Writing – original draft:** Zhijun Xie.

**Writing – review & editing:** Zhijun Xie, Wenfu Tang.

## Supplementary Material


